# Three-Dimensional Avian Hematopoietic Stem Cell Cultures as a Model for Studying Disease Pathogenesis

**DOI:** 10.3389/fcell.2021.730804

**Published:** 2022-01-20

**Authors:** Vladimir Zmrhal, Andrea Svoradova, Andrej Batik, Petr Slama

**Affiliations:** ^1^ Department of Animal Morphology, Physiology and Genetics, Faculty of AgriSciences, Mendel University in Brno, Brno, Czech Republic; ^2^ NPPC, Research Institute for Animal Production in Nitra, Luzianky, Slovak Republic

**Keywords:** bone marrow niche, disease prevention, hematopoietic stem cell, poultry, three-dimensional cell culture

## Abstract

Three-dimensional (3D) cell culture is attracting increasing attention today because it can mimic tissue environments and provide more realistic results than do conventional cell cultures. On the other hand, very little attention has been given to using 3D cell cultures in the field of avian cell biology. Although mimicking the bone marrow niche is a classic challenge of mammalian stem cell research, experiments have never been conducted in poultry on preparing *in vitro* the bone marrow niche. It is well known, however, that all diseases cause immunosuppression and target immune cells and their development. Hematopoietic stem cells (HSC) reside in the bone marrow and constitute a source for immune cells of lymphoid and myeloid origins. Disease prevention and control in poultry are facing new challenges, such as greater use of alternative breeding systems and expanding production of eggs and chicken meat in developing countries. Moreover, the COVID-19 pandemic will draw greater attention to the importance of disease management in poultry because poultry constitutes a rich source of zoonotic diseases. For these reasons, and because they will lead to a better understanding of disease pathogenesis, *in vivo* HSC niches for studying disease pathogenesis can be valuable tools for developing more effective disease prevention, diagnosis, and control. The main goal of this review is to summarize knowledge about avian hematopoietic cells, HSC niches, avian immunosuppressive diseases, and isolation of HSC, and the main part of the review is dedicated to using 3D cell cultures and their possible use for studying disease pathogenesis with practical examples. Therefore, this review can serve as a practical guide to support further preparation of 3D avian HSC niches to study the pathogenesis of avian diseases.

## Introduction

As a precursor of immune cells, hematopoietic stem cells (HSC) play an indispensable role in the immune response against the main avian diseases (Cui et al., 2009; Gurung et al., 2017; Hosokawa et al., 2020). HSC reside in a unique bone marrow environment, where they interact with other cells and molecules to create a bone marrow niche (Zhang P. et al., 2019). Hematopoietic colonization of the bone marrow starts at 13 days of embryonic development. During embryonic life, the bone marrow, yolk sac, and liver contribute temporarily to hematopoiesis. Subsequently, the bone marrow serves as the major site for hematopoiesis in adult chickens (Guedes et al., 2014). HSC fate, migration, and differentiation are influenced by many factors, and these circumstances complicate the preparation of realistic *in vitro* HSC culture. Current approaches in mimicking bone marrow environments comprise scaffold-based systems using hydrogels, and macroporous and nanofiber scaffolds (Bello et al., 2018). These scaffolds can be embedded in perfused chambers and through microfluidic technology, further enhancing the biocompatibility of cell culture (Bhatia and Ingber, 2014). Nowadays, increasing interest is given to using these culture systems to study interactions of immune cells with bacteria and other pathogens (Lee et al., 2021). *In vitro* studies constitute the gold standard for researching disease pathogenesis; it is interesting that there exists a lack of studies involving pathogens' interactions with HSC to evaluate impacts of disease causative agents on the fate and differentiation of HSC and on immune cell development. In human medicine, relatively many studies have been conducted to describe immunosuppressive mechanisms of human viruses in three-dimensional (3D) cell culture, as reviewed by He et al. (2016). In chickens, there have been no experiments with 3D cell culture to study disease pathogenesis. The main goal of this review, therefore, is to provide information about 3D cell cultures that will be useful for *in vivo* avian HSC 3D culture preparation and further conceiving of host–pathogen studies.

## Hematopoietic Stem Cells and Hematopoiesis

**FIGURE 1:**
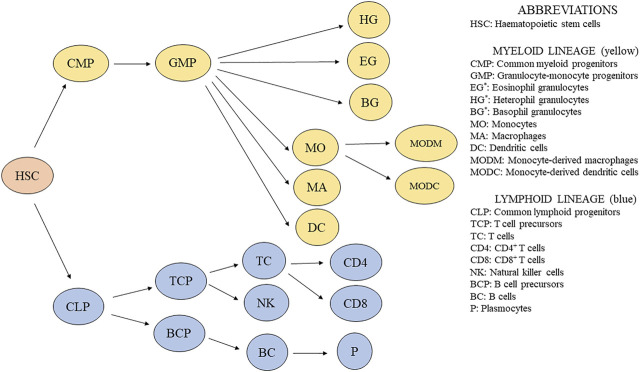
Simplified scheme of immune cell development in chickens. ^*^Granulocytes are usually distinguished by light microscopy using blood smears, and their percentage is counted. Additionally, granulocytes are recognized based on their granularity by flow cytometry (Bilkova et al., 2017).

Hematopoiesis is a group of processes giving rise to blood cells. The para-aortic foci comprise a source of HSC in embryos. From there, HSC colonize the developing lymphoid organs (Yvernogeau and Robin, 2017). CD45^+^ progenitors of immune cells from the bone marrow colonize the bursa of Fabricius, thymus, and spleen already during embryonic development and continue in this function throughout the life of the bird (Fellah et al., 2014). A simplified scheme showing the development of avian immune cells is shown in Figure 1.

Growth factors and cytokines stimulate immune cell differentiation from hematopoietic progenitors, so they can be used *in vitro* for cell differentiation studies. Stem cell factor (SCF) is the main cytokine responsible for the self-renewal, proliferation, and differentiation of stem cells and their progenitors. SCF is a ligand for c-kit receptor (Siatskas and Boyd, 2000).

Differentiation of myeloid lineage is ensured by colony-stimulating factors (CSFs). Macrophage CSF elicits macrophage differentiation from bone marrow progenitors. The same effect has been shown in IL-34 (Garceau et al., 2015; Wu et al., 2020). Granulocyte CSF stimulates monocyte growth and mobilizes heterophils from the bone marrow when inflammation occurs (Kogut et al., 1997; Gibson et al., 2009). The final member of the CSF family in chickens is granulocyte-macrophage CSF, which by itself is responsible for macrophage differentiation from monocytes (Peng et al., 2020) as well as the proliferation of tissue macrophages and increasing responsiveness to macrophage CSF (Chen et al., 1988). Together with IL-4, granulocyte-macrophage CSF can stimulate dendritic cells differentiation from bone marrow progenitors (Wu et al., 2010) and blood monocytes (Kalaiyarasu et al., 2016). Chicken myelomonocytic growth factor has a function similar to that of CSF because it stimulates proliferation and differentiation of granulocytes and macrophages *in vitro* (Siatskas and Boyd, 2000). The chicken homolog of CAAT, NF-M, has a proven ability to stimulate the differentiation of eosinophils in the bone marrow (Müller et al., 1995).

T-cell colonization of the thymus begins in waves during embryonal development when HEMCAM+ and c-kit+ bone marrow precursors colonize the cortex of the thymic lobules (Vainio et al., 1996). Both beta 2-microglobulin (Dunon et al., 1990) and CCL21 chemokine (Annamalai and Selvaraj, 2010; Kozai et al., 2017) seem to be involved in this process. Additional differentiation of naïve T cells occurs under the regulation of cytokines based on immune response against pathogens, as reviewed by Kogut (2000) and Wigley and Kaiser (2003).

B-cell precursors from the bone marrow colonize bursal anlage from 10 days of embryogenesis (Mansikka et al., 1990). Based on the expression of CXCR4 on B cells and their precursors, it can be assumed that CXCL12 chemokine plays a role in the migration of B-cell precursors (Nagy et al., 2020). Interestingly, B-cell precursors can be found in the bone marrow during embryogenesis but not after hatching, presumably because the colonization of bursal anlage by the bone marrow precursor occurs only in embryos (Weber and Foglia, 1980). In a more recent study, B-cell precursors were sorted based on cell size, and larger precursors were observed to proliferate and differentiate during development more than did smaller precursors (Ko et al., 2018). After antigen stimulation, naïve B cells can differentiate into plasmocytes and stimulate the production of specific antibodies. B-cell biology in chicken has been reviewed in detail by Sayegh et al. (2000). Relevant distinctive markers of avian immune cells for phenotype analysis are shown in Table 1.

**TABLE 1:** Distinctive markers of chicken immune cells derived from hematopoietic stem cells.

Type of cell	Markers with references
Hematopoietic stem cells	CD45 (Hao et al., 2020), HEMCAM (Lampisuo et al., 1998), c-Kit (Yvernogeau and Robin, 2017)
Common myeloid progenitors	CSF1R (Garcia-Morales et al., 2014), c-Kit (Yvernogeau and Robin, 2017)
Granulocyte-monocyte progenitors	CSF1R (Garcia-Morales et al., 2014)
Heterophil granulocytes	MMP9, MRP126, LECT2, CATHL1, LYG2, LYZ, RSFR (Sekelova et al., 2017)
Eosinophil granulocytes	MEP17, EOS47 (Yvernogeau and Robin, 2017)
Monocytes	CD11c, MRC1L-B (Hao et al., 2020), CSF1R (Garcia-Morales et al., 2014)
Macrophages	MRC1L-B (Hao et al., 2020), CSF1R (Garcia-Morales et al., 2014)
Dendritic cells	Dendritic cells: CD11c (Hao et al., 2020), CSF1R, MHC-II (Nagy et al., 2016), CD83 (Kalaiyarasu et al., 2016)
Monocyte-derived macrophages	MRC1L-B, MHC-II (Peng et al., 2020)
Monocyte-derived dendritic cells	CD83, MHC-II (Kalaiyarasu et al., 2016)
Common lymphoid progenitors	HEMCAM (Lampisuo et al., 1998), c-Kit (Yvernogeau and Robin, 2017)
T-cell precursors	CD3 (Chen et al., 1994), HEMCAM, chL12, c-kit (Lampisuo et al., 1998)
T cells	CD3, CD8, CD4 (Hao et al., 2020)
Natural killer cells	CD8α (Hao et al., 2020), CD107 (Jansen et al., 2010)
B-cell progenitors	CXCR4 (Nagy et al., 2020), Bu-1 (Lampisuo et al., 1998)
B cells	Bu-1 (Hao et al., 2020), CXCR4 (Nagy et al., 2020)
Plasmocytes	CD57 (Mast and Goddeeris, 1998)

### Avian Bone Marrow

In birds, the bone marrow distribution is highly correlated with the existence of medullary bones; therefore, the major sites of bone marrow hematopoiesis in adult birds are the femur and tibiotarsus (Canoville et al., 2019). Brandon et al. (2000) optimized conditions to obtain a full range of blood cells from hematopoietic precursors in quail. They isolated bone marrow cells by gradient centrifugation and separated adherent cells by overnight adherence selection. Obtained blast-like cells were cultured on methylcellulose and fibrin gel cultures and differentiated in 6 morphologically different colonies. The most abundant were granulocyte-macrophage, erythroid, and macrophage cell colonies. After 6 days of culture, cells achieved maximal differentiation, and May–Grunwald–Giemsa staining was performed to recognize cells phenotype. Based on the visual evaluation, erythroblasts, erythrocytes, aggregated macrophages, monocytes, heterophils, basophils, eosinophils, and thrombocytes were recognized; therefore, they successfully imitate the avian hematopoiesis *in vitro*. Additionally, Nazifi et al. (1999) provided a detailed composition of quail bone marrow. Erythroid cells were the most prevalent (almost 70%), and myeloid cells comprised 25% of the whole cell population. Myeloid/erythroid ratios were described in various avian species, such as 1 for ducks (Tadjalli et al., 1997), 1.24 for pheasants (Tadjalli et al., 2013), and 1:14.6 for chickens at 4 weeks (Glick and Rosse, 1981). Glick (1987) showed that cellular composition is constant in chicken femur and tibiotarsus, but with increasing age, the numbers of immature granulocytes decreased. Based on light microscopy evaluation, the cellular composition of the bone marrow from various avian species was described, including chicken (Glick, 1987), partridge (Tadjalli et al., 2012), duck (Tadjalli et al., 1997), black-headed gull (Tadjalli and Hadipoor, 2002), pheasant (Tadjalli et al., 2013), Japanese quail (Nazifi et al., 1999), and parrots (Schwartz et al., 2019).

### Bone Marrow Hematopoietic Stem Cell Niche

**FIGURE 2:**
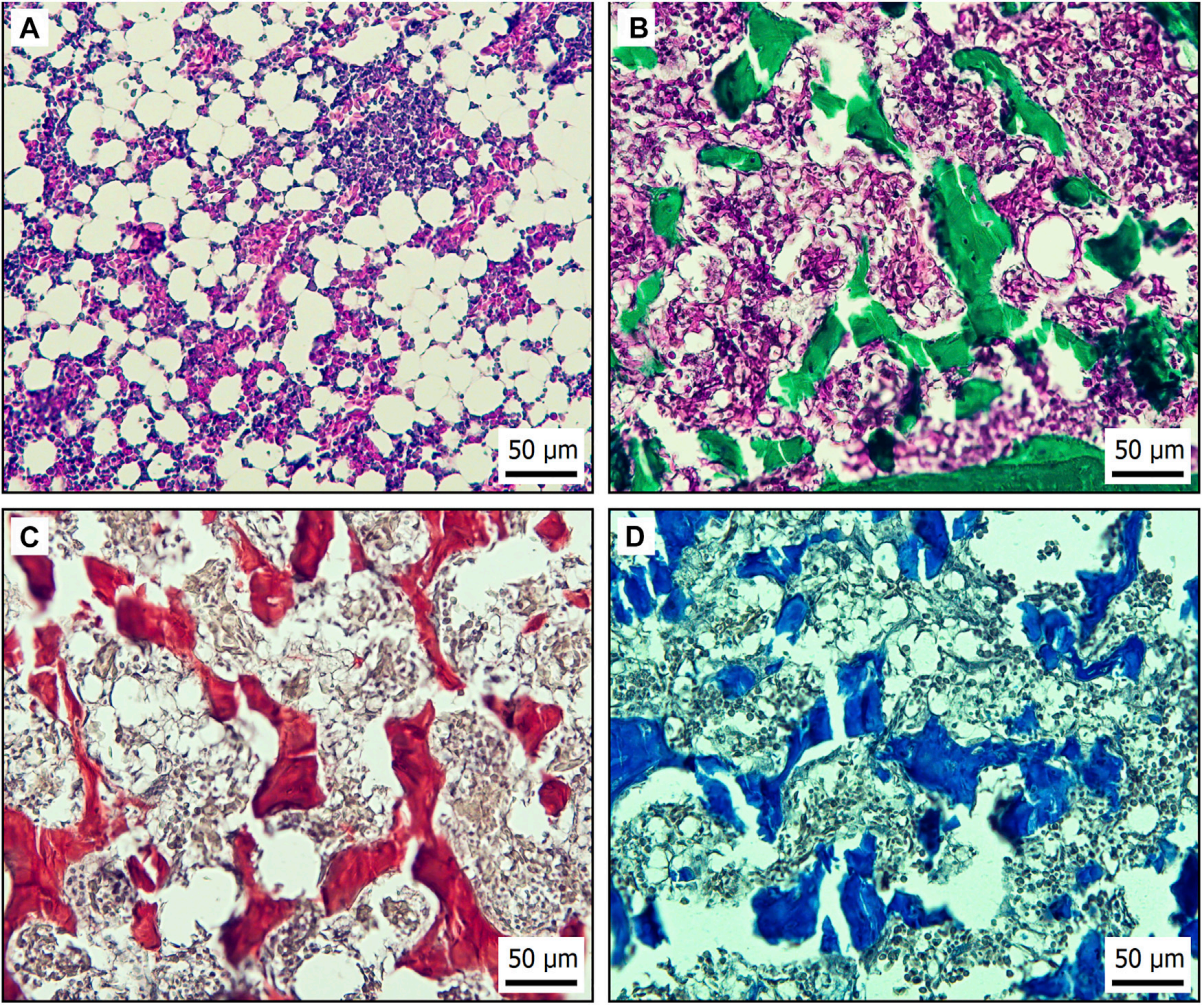
Histological bone marrow specimens from laying hen aged 21 weeks. **(A)** H&E staining of vascular niche in bone marrow. **(B)** Endosteal region of bone marrow stained by Masson’s green trichrome to demonstrate connective tissue based on collagen (green). **(C)** Sirius red staining of collagen type I (red) in endosteal niche of bone marrow. **(D)** Endosteal niche of bone marrow stained by Alcian blue to recognize hyaluronic acid (blue) in extracellular matrix.

In order properly to imitate the bone marrow environment, it is necessary to understand the morphological structure of the avian HSC bone marrow niche. Two main subniches can be distinguished in the bone marrow. The endosteal niche is located close to the osteoblasts in the endosteum (Bello et al., 2018). In the endosteal niche, HSC prevalently reside in a quiescent state due to high concentrations of Ca^2+^ and hypoxia. The second subniche is termed the vascular niche (Figure 2A) where, by contrast, HSC can easily proliferate and differentiate because there is a higher level of oxygen and lower concentration of Ca^2+^ in comparison with the endosteal niche (Ferreira and Mousavi, 2018). Niche physical properties strongly influence HSC fate. Young’s modulus (YM) is an indicator of materials’ stiffness. In the case of the endosteal region, values of about 40–50 kPa have been determined, but areas near the blood vessels are much softer, with YM < 3 kPa (Shrestha and Yoo, 2019). Because greater tissue stiffness directly impairs HSC differentiation, in an endosteal niche, HSC are losing their stemness (Wen et al., 2014). In the endosteal niche, HSC reside in a quiescent state that is necessary for sustaining long-term hematopoiesis. Through signaling and adhesion molecules, osteoblasts regulate quiescence in HSC and ensure maintenance of quiescent HSC (Arai and Suda, 2007).

Quiescent HSC from the endosteal niche eventually travel through the vascular niche, where they undergo differentiation and expansion. The vascular niche allows HSC to enter the peripheral blood through blood vessels (Tamma and Ribatti, 2017). More specifically, the vascular niche is divided into arterial and sinusoidal niches (Zhang P. et al., 2019). In avian species, CD45^+^ HSC have been described outside the bone marrow sinuses in extravascular regions near the arteries, where lymphopoiesis and myelopoiesis occur (Olah et al., 2014). Many cell types influence HSC fate within the (sub)niches, but some of these seem to be more important in creating those (sub)niches. Endothelial cells line the border of arteries and express high levels of VCAM-1 adhesion molecule, which ensures retention of HSC (Ulyanova et al., 2005). On the other hand, HSC homing and proliferation are supported by E selectin molecules expressed on sinusoidal endothelial cells (SEC) (Winkler et al., 2012). Mesenchymal stem cells (MSC), often termed mesenchymal stromal cells, play an important role in creating a stroma for the bone marrow niche and influencing HSC by cell-to-cell contact and production of active molecules (Walenda et al., 2010; Walenda et al., 2011). Coculture of HSC and MSC in collagenous hydrogel has revealed some important findings. HSC were observed to have a higher capacity for self-renewal in 3D cell culture, and coculture with MSC further supports HSC proliferation. MSC produce fibronectin and osteopontin that form an extracellular matrix (ECM) component, which, in turn, allows HSC easier migration due to the presence of cell attachment sites (Leisten et al., 2012). MSC further produce IL-6, granulocyte-macrophage CSF, SCF, and adhesion molecules such as VCAM-1 and E-selectin. By means of all these molecules, MSC regulate homing, proliferation, and differentiation of HSC (Li and Wu, 2011). Chicken MSC express CD73 and CD44 and can easily be isolated from the bone marrow and used to coculture with HSC to mimic HSC niche environment (Adhikari et al., 2019).

Moreover, an HSC niche is composed of other cellular components that produce molecules involved in HSC self-renewal, quiescence, or proliferation. Endothelial cells are further divided into SEC and arteriolar endothelial cells (AEC), and the biggest difference between them is in the production of SCF, which ensures the maintenance of HSC. AEC are more effective producers of SCF than SEC (Ding et al., 2012; Xu et al., 2018). The rich population of bone marrow adipocytes is another producer of SCF, which plays an indispensable role in the maintenance of HSC (Zhou et al., 2017). Megakaryocytes give rise to thrombocytes in blood. On the other hand, they directly influence hematopoiesis by cytokine production. They can affect HSC in various ways. Firstly, megakaryocytes ensure quiescence of myeloid-based HSC in megakaryocyte-dependent regions by the production of CXCL4 (Pinho et al., 2018) and TGF-β (Gong et al., 2018). Secondly, megakaryocytes can promote the proliferation of HSC by the production of fibroblast growth factors (Zhao et al., 2014). TGF-β is known as the inducer of quiescence, but it was proven that TGF-β in low concentration can promote the proliferation of myeloid HSC. However, the quiescence-promoting effect of a high concentration of TGF-β is undisputed (Blank and Karlsson, 2015). Various cell types are further involved in TGF-β production, for instance, Schwann cells (Yamazaki et al., 2011) and macrophages (Hur et al., 2016). Interferons (IFN-α, IFN-γ) are known inducers of an antiviral state in a cell, but they can cause loss of quiescence and promote the proliferation of HSC. TNF-α as a major pro-inflammatory cytokine was found to suppress proliferation of HSC by induction of apoptosis, but some studies described positive effects of TNF-α treatment on the expansion of lymphoid progenitors and bone marrow granulocytes; therefore, the influence of TNF-α on HSC fate is controversial (Baldridge et al., 2011).

Various immune cells influence HSC in direct or indirect ways, and these cells are the target for most pathogens. Therefore, there is another way in which pathogens influence HSC in their niche. The most known influencers of HSC are regulatory T cells and subtypes of macrophages (Man et al., 2021). Marek’s disease in susceptible chicken lines triggers the production of TGF-β^+^ regulator T cells, and subsequently high amounts of TGF-β released into the bloodstream dysregulate hematopoiesis (Gurung et al., 2017). The same CD4^+^ CD25^+^ regulatory T cells are induced in cecal tonsil of *Salmonella*-infected chickens (Shanmugasundaram et al., 2015). Regulatory T cells, via the production of adenosine, can ensure further quiescence of allogeneic HSC (Hirata et al., 2018). Many viral pathogens replicate in bone marrow macrophages (von Bülow and Klasen, 1983a), and these phagocytic cells influence HSC in a direct way. They can provide retention sites for HSC through VCAM-1 (Dutta et al., 2015) and induction of expression of CXCL12 in MSC (Chow et al., 2011). Therefore, bone marrow macrophage’s function seems to be promoting retention of HSC by regulating osteoblast and MSC to maintain retention of HSC, because treatment with granulocyte CSF caused rapid depletion of osteoblast and endosteal region macrophages. Due to the loss of binding sites and factors responsible for the retention, high numbers of HSC were released into the blood (Winkler et al., 2010).

Non-cellular HSC niche components such as ECM proteins not only play supporting roles but also have the ability to greatly influence HSC functions. Furthermore, they can be easily incorporated into various 3D cell culture scaffolds (Caliari and Burdick, 2016). The most abundant molecules in HSC niches are proteoglycans, which ensure signal delivery between cells (Redondo et al., 2017) and fibrous proteins (Figure 2B) (Frantz et al., 2010). Collagen I, a major ECM fibrous protein of the bone marrow, causes a decrease in CD34^+^ HSC expansion (Oswald et al., 2006) and an increase in the numbers of cells in a quiescent state. That is because collagen I is associated with the endosteal region (Figure 2C), where HSC quiescence occurs (Chitteti et al., 2015). Also in the endosteal region, hyaluronic acid supports osteogenesis while ensuring viscoelasticity and compressive strength of the bone marrow (Figure 2D). Moreover, hyaluronic acid provides attachment sites through ICAM-1 and CD44 expressed on chicken MSC (Adhikari et al., 2019; Zhai et al., 2020). The additional fibrous proteins fibronectin and laminin express tri-amino acid sequence (arginine–glycine–aspartate) and are termed RGD peptides (Bellis, 2011). Integrins are RGD peptide ligands with numerous functions in HSC (Coulombel et al., 1997). For instance, β7 integrin activation has been found to regulate HSC homing and engraftment (Murakami et al., 2016). HSC in a presence of laminin and fibronectin within *ex vivo* culture improved bone marrow engrafting ability and stem cell expansion, probably due to integrin-induced pathways (Sagar et al., 2006).

The Notch signaling pathway is involved in the proliferation of HSC. Through stimulation of Notch receptors, expansion of HSC in the niche can be achieved (Mendelson and Frenette, 2014). On the other hand, the Wnt signaling pathway is involved in HSC proliferation and differentiation based on the level of activation. Only mild activation of the Wnt pathway increased HSC proliferation. Subsequently, by increasing Wnt activation, differentiation into myeloid precursors and then into lymphoid precursors occurs. If the Wnt pathway is highly stimulated, hematopoiesis impairment occurs (Luis et al., 2011).

An HSC niche is a complex system, where many factors affect HSC function. Because specific information about chicken bone marrow is lacking in this area, it is necessary to apply information about mammalian HSC niches to the preparation of an *ex vivo* chicken HSC niche.

### Ensuring Quiescence in *In Vitro* Studies

In *in vitro* models, achieving the quiescent and active states of HSC is critical for studying the process of hematopoiesis. In a recent study, Kobayashi et al. (2019) defined key factors responsible for maintaining a quiescent state *in vitro* HSC cultures. The first factor is low oxygen concentration, which is about 1.3% (Spencer et al., 2014). In mice, it was found that over 80% of quiescent HSC had low adenosine triphosphate levels and utilize cytoplasmic glycolysis (Simsek et al., 2010). Secondly, attenuation of metabolic processes necessary for maintaining quiescent stadium is achieved by relatively high fatty acid concentration (Kobayashi et al., 2019). Lastly, the crucial factor is low cytokines concentration because it is well known that cytokines and growth factors ensure mobilization and activation of HSC (Jahandideh et al., 2020). Under these conditions, Kobayashi et al. (2019) were able to maintain engraftable quiescent HSC for 1 month. To study the influence of pathogens on activation of HSC and further differentiation, it must be achieved by changes during the HSC culture, which can be ensured by perfusion systems with the flow of the medium. Furthermore, the system must allow the incorporation of nutrients and cytokines. 3D HSC culture system prepared by Rödling et al. (2017) was a bioreactor filled with macroporous hydrogel with niche-mimicking properties, and the flow of the medium was powered by a peristaltic pump. Their attention was focused on mimicking steady-state and activation conditions in HSC culture. They measured cytokine levels in static and dynamic cultures, and it is not surprising that levels of cytokines were much lower in dynamic culture because they washed out the 3D culture. In static culture, strong upregulation of IGFBP2 and MIF cytokines was found, and both were described to support proliferation and expansion of HSC. Hypoxia in cell culture can be easily achieved by packing the cell culture chambers with vacuum-sealing machines (Matthiesen et al., 2021). In hypoxic cells is a characteristic high expression of hypoxia-inducing factor (HIF), and quiescent HSC express high levels of HIF-1α (Takubo et al., 2010).

### Impacts of Avian Immunosuppressive Diseases on Bone Marrow-Derived Cells

Most diseases cause immunosuppression because disease agents must overcome the organism’s immune barrier. *In vitro* studies in this area generally have been conducted with individual bone marrow-derived cells, but we lack information about direct impacts on HSC and hematopoiesis as a whole. A better understanding of immunosuppression is important for determining better preventive and diagnostic mechanisms for avian diseases (Gimeno and Schat, 2018). Avian immunosuppressive diseases have been described in detail in reviews by Schat and Skinner (2014) and by Gimeno and Schat (2018), so we have narrowed our focus to summarizing information about avian diseases and their impacts on bone marrow-derived cells cultured in cell culture wells and flasks. To date, a methodology for generating cells from the bone marrow in chickens has been described only for dendritic cells (Wu et al., 2010). Therefore, most studies in this area have been focused on the main antigen-presenting cells (Table 2).

**TABLE 2:** Effects of avian diseases on avian bone marrow-derived cells in *in vitro* experiments.

Cell type	Causative agent	Effect of pathogen on cells
Bone marrow mononuclear cells	ALV-J	Inhibition of differentiation into DC and maturation of DC. Induction of apoptosis. Reduction of TLR1, TLR2, TLR3, MHC-I, and MHC-II expression in surviving DC (Liu et al., 2016a)
Bone marrow mononuclear cells	CAV	Infection of cells from chicks 6 days old but decreasing numbers of infected cells in chicks 28 days old. Increasing replication in cells until 48 h post-infection (McNeilly et al., 1994)
Mesenchymal stem cells	IBDV	Increasing replication of IBDV in MSC 6, 24, 48, and 72 h post-infection (Khatri and Sharma, 2009)
Macrophages	MDV, HVT, IBV, REV, Adenovirus, ILV, reovirus, IBDV, NDV	Adenovirus, ILV, reovirus, IBDV, and NDV were found to replicate in MA and change their morphology. MA were resistant against MDV, herpesvirus of turkeys (HVT-FC126), IBV, and REV (von Bülow and Klasen, 1983a)
Macrophages	MDV	Lymphokine-activated MA caused growth inhibition of MDV T-lymphoblastoid cell line (von Bülow and Klasen, 1983b)
Dendritic cells	AIV H9N2	Upregulation of genes involved in signal transduction, transmembrane transport, and inflammatory responses. Downregulation of genes involved in metabolic processes and MHC-I antigen presentation (Liu et al., 2020)
Dendritic cells	Pustulan (C type lectin ligand)	Pustulan induced the same expression of MHC-II and pro-inflammatory cytokines as did IBV. Moreover, pustulan induced CD4^+^ T-cell response against IBV (Larsen et al., 2020)
Dendritic cells	IBDV	Increase in CD40 and CD86 expression. Stimulation of CD4^+^ lymphocytes (Liang et al., 2015)
Dendritic cells	LPAI, HPAI	LPAI H5N2 caused rapid increase in IFN-α/β expression. Together, HPAI H5N2 and H7N1 caused upregulation of IL-8, IFN-α, and IFN-γ and of TLR3 and TLR21 (Vervelde et al., 2013)
Dendritic cells	*Salmonella enteritidis*	Increased expression of CD40, CD80, and MHC-II molecules and of IL-6 and IL-12 cytokines (Kamble et al., 2016a)
Dendritic cells	IBDV	Increased expression of CD86 and MHC-II. Slightly higher apoptosis and necrosis levels after IBDV activation. Higher production of Th1 cytokines IFN-γ and IL-12α and of TLR3 (Yasmin et al., 2015)
Dendritic cells	*Salmonella enterica* serovar Gallinarum	Elevating expression of IL-6, IL-10, and IFN-γ in stimulated DC and production of IL-2 in coculture with CD4^+^ T cells (Kamble et al., 2016b)
Dendritic cells	IBDV	Genome-wide profiling with upregulating genes involved in oxidative phosphorylation, T cell receptor, and IL-17 signaling pathways (Lin et al., 2016)
Dendritic cells	Velogenic and lentogenic strains of NDV	Higher capacity of velogenic strain to replicate in lipopolysaccharide-activated DC. Velogenic strain caused stronger cytokine production than did lentogenic strain (Xiang et al., 2018)
Dendritic cells	ALV-J	Infection of DC in early phases of differentiation and induction of apoptosis by disruption of nutrient processing and metabolic function (Liu et al., 2016b)
Dendritic cells	*Lactobacillus johnsonii*	Increased expression of CD40, CD86, and MHC-II molecules; IL-12, IFN-γ, IL-1β, and IL-6 cytokines; and CXCLi1 and CXCLi2 chemokines. Upregulation of TLR2 and TLR5 expression (Huang et al., 2020)

Note. AIV, avian influenza virus; ALV-J, avian leucosis virus-J; CAV, chicken anemia virus; DC, dendritic cells; HPAI, highly pathogenic avian influenza; HVT, herpesvirus of turkeys; IBDV, infectious bursal disease virus; IBV, infectious bronchitis virus; IFN, interferon; ILV, infectious laryngotracheitis virus; LPAI, low pathogenic avian influenza; MA, macrophages; MDV, Marek’s disease virus; MHC, major histocompatibility complex; MSC, mesenchymal stem cells; NDV, Newcastle disease virus; REV, reticuloendotheliosis virus; TLR, toll-like receptor.

### Deriving Chicken Stem Cells From Bone Marrow

Standard methods for avian stem cells culture are described in a review by Farzaneh et al. (2017). Derivation of HSC niche cells is the first step in cell culture preparation. In chickens, methods have been described for isolating HSC and MSC. In the case of avian HSC, the usual sources are chicken femur and tibiotarsus. The ends (epiphysis) of the bones are cut, and their content is flushed with sterile Dulbecco’s phosphate-buffered saline without Ca and Mg (DPBS). Cell aggregates in the flushed content of the bones must be disaggregated by pipetting and sieved through a 40-μm cell strainer. Subsequently, bone content diluted in DPBS is loaded onto an equal volume of Histopaque®-1,119 (1.119 g/ml at 25°C) and centrifuged at 1,200 *g* for 30 min. Cells at the interface are collected and then washed two times with PBS. Cells are cultured in complete RPMI-1640 medium with 10% of chicken serum at 41°C and 5% CO_2_ (Wu et al., 2010). Anti-chicken CD45 phycoerythrin (PE)-conjugated antibody (SouthernBiotech, Birmingham, AL, USA) can be used in combination with anti-PE microbeads (Miltenyi Biotech, Bergisch Gladbach, Germany) to isolate HSC by magnetic-activated cell sorting.

MSC can be embedded simultaneously with HSC into 3D cell culture to improve the biocompatibility of the culture. MSC can be isolated from compact bones of the femurs and tibiotarsus of day-old chicks. The bone marrow must be completely washed out, and bones are then chopped into small pieces. Bone fragments are disaggregated in Dulbecco’s modified Eagle’s Medium (DMEM) with 0.25% collagenase and then incubated in a shaking bath for 60 min at 37°C and 180 rpm. Suspension with bone fragments is filtered through a 40-μm cell strainer. Subsequently, the cell suspension is washed in DMEM, then placed in complete DMEM, and transferred into a humidified incubator at 37°C and 5% CO_2_. After 24 h of incubation, non-adherent cells are removed, and adherent cells are considered as MSC (Adhikari et al., 2019; Adhikari et al., 2020).

### Three-Dimensional Cell Cultures

*In vitro* studies are the gold standard for studying various cell types and for understanding the pathogenesis of diseases. Classic culture using plastic culture wells or dishes, where cells have adhered to the surface, does not imitate cell niche microenvironment inside the body. Very little attention has been given to the use of 3D cell cultures in avian models. It has been studied predominantly in embryonal cells, such as primordial germ cells (Chen et al., 2018), intestinal epithelial cells (Pierzchalska et al., 2019), or chicken bone marrow cells as support for osteogenesis of human stromal cells (Yang et al., 2020). Therefore, our current knowledge about 3D stem cell cultures comes from studies in mammals. An *in vitro* HSC niche cell culture model has never been studied in avian species.

Two-dimensional (2D) cell culture has some disadvantages, as explained by Kapałczyńska et al. (2018). Such cell cultures do not imitate the natural structure of tissue and do not provide cell-to-cell and cell-to-microenvironment interactions. Adherence to the plastic surface causes cells to take on an unnatural flattened shape and changes their morphology. In contrast to tissue, cells in 2D cultures have unlimited access to oxygen, nutrients, metabolites, and signaling molecules. Finally, 2D cell cultures cause changes in gene expression and topography of the cells in comparison with the *in vivo* environment.

### Hydrogel Scaffolds

All limitations of 2D cell cultures hinder the study of cell proliferation and differentiation in ways that are similar to doing so in tissue. For these reasons, scaffolds based on hydrogels have been developed for 3D cell culture, and the scaffolds should mitigate these limitations. Caliari and Burdick prepared a practical, focused review as a hydrogel selection guide (Caliari and Burdick, 2016).

Three-dimensional HSC culture requires biocompatible materials that ensure imitation of bone marrow niche elements as influencers of HSC fate. ECM of the bone marrow is composed of the most prevalent collagen structural proteins (Kramer et al., 2017) and then the important laminin and fibronectin proteins (Zhang P. et al., 2019) that are necessary for the growth, development, proliferation, and differentiation of HSC. These proteins express integrin-binding domains termed RGD. HSC express on their surfaces such RGD-binding integrins as α4, α6, α7, α9, and β1 that play important roles in cell development and proliferation (Zhang P. et al., 2019). Collagen I, collagen IV, laminin, and fibronectin together have been able significantly to increase HSC expansion and induce greater myeloid progenitor cell expansion (Celebi et al., 2011). Not only ECM but also niche cells contribute to the production of molecules that build a hematopoietic niche. The MSC-based scaffold has been shown to provide signaling molecules for HSC, because αIIb, αV, and β3 integrins were induced in HSC within decellularized ECM scaffolds derived from SCP-1+ MSC. These findings support the contribution of MSC to the modulation of HSC function by cell-to-cell contact (Kräter et al., 2017).

Leisten et al. (2012) performed a coculture of HSC with MSC in collagen-based hydrogel and revealed some useful findings. A collagenous matrix is optimal for HSC migration and proliferation. Proliferation marker Ki67 was highly expressed, suggesting high self-renewal in HSC. MSC in coculture produce fibronectin and osteopontin, and they support collagen I synthesis to form a hematopoietic niche. MSC isolated from the bone marrow in collagen-based hydrogel stimulated higher levels of CD34^+^ HSC. This ability was proven only in the case of MSC derived from the bone marrow, so coculture with bone marrow-derived HSC is necessary for the applicability of bone marrow MSC (Leisten et al., 2012). Collagen-based hydrogels seem to be a promising platform for HSC 3D cell culture, but naturally based hydrogels have shortcomings in terms of their mechanical properties. These hydrogels have limitations with regard to controlling the material stiffness and elasticity. Holst et al. (2010) found that the addition of tropoelastin, which changed stiffness and elasticity, caused greater expansion of hematopoietic progenitor cells. For this reason, synthetic hydrogels with easier controllability of stiffness have been developed.

Polyacrylamide (PAM) has a uniquely tunable mechanical character that allows setting up optimal stiffness and elasticity (Kandow et al., 2007). As indicated by YM, tensile stiffness of the bone marrow has been established to be in the range 0.25–24.7 kPa at the physiological temperature (Jansen et al., 2015). In a recent study with ECM ligand-coated PAM, it was suggested that biophysical elements combined with ligands (laminin or fibronectin) of the HSC niches directly modulate HSC fate decisions (Choi and Harley, 2017).

Synthetic hydrogels based upon polyethylene glycol (PEG) present another choice for culture with tunable stiffness (Carthew et al., 2018). The biggest advantage of PEG is its enormous biocompatibility (Tsou et al., 2016). Macroporous PEG-based hydrogel is indicated as a great approach for mimicking an HSC niche because it reflects 3D bone marrow regions. Coculture with bone marrow-derived MSC in PEG-based hydrogel led to the much higher proliferation and self-renewing capacity of HSC (as in the natural environment) compared with ordinary 2D cell culture (Raic et al., 2014). PAM- and PEG-based hydrogels or scaffolds do not provide cell adhesion and support proliferation, but this disadvantage can easily be overcome by conjugation with RGD peptides. This treatment ensures MSC (Sawyer et al., 2005) and HSC adhesion and proliferation (Raic et al., 2014; Tsou et al., 2016).

Hydrogels with synthetic crosslinkers can be prepared based on hyaluronic acid, which allows for easier stiffness tunability. On the other hand, fully synthetic hydrogels are composed of chemically defined components that ensure easy stiffness tunability, administration of adhesive proteins, and cell recovery, but biocompatibility is lower (Tibbitt and Anseth, 2009). The main components of these hydrogels consist of polymers, such as polyvinyl alcohol or dextran with crosslinkers such as PEG that connect polymer chains (Caliari and Burdick, 2016). Hyaluronic acid-based hydrogels can be enriched with carbon nanotubes to ensure antioxidant properties, thereby supporting HSC proliferation and pluripotency and protecting against oxidative stress (Zhang Y. et al, 2019). Results from testing these hydrogels in mammalian models show that this technique is promising for application in avian models. Host–pathogen interactions can be conducted simply on hydrogels embedded in cell culture. The hydrogels also can be included in advanced cell culture systems, such as organoids and organ-on-a-chip (OCM), where interactions with pathogens can be studied (Liu et al., 2019; Feaugas and Sauvonnet, 2021).

### Nanofiber Scaffolds

Several synthetic materials have been used to mimic the bone marrow niche. Among these are polycaprolactone (PCL), polylactic acid, polyurethane, and polyethylene terephthalate. These polymers have several limitations, including lower biocompatibility, hydrophobicity (compared with cells niche), and lack of binding sites for cell adhesion (Ferreira and Mousavi, 2018). On the other hand, synthetic materials have good mechanical properties, are highly reproducible, can be produced at a low cost, and do not stimulate the activation of immune cells (Tallawi et al., 2015; Kim T. E. et al., 2016).

The absence of biocompatibility and binding sites can be overcome relatively easily by blending polymers with ECM proteins and coating them with adhesion molecules. Ma et al. (2008) blended poly(dl-lactide-*co*-glycolide) polymer with collagen I to create nanofibers by electrospinning method and then coated nanofibers with E-selectin. This nanofiber scaffold increased HSC capture by 44% within 30 min and by 40% within 60 min. Biocompatibility can be increased by the inclusion of other HSC niche cells. HSC coculture with MSC on poly-l-lactic acid nanofiber scaffold led to greater expansion, purity, viability, and clonogenicity of HSC (Darvish et al., 2019).

PCL is widely used in tissue engineering and drug delivery (Chen and Lin, 2020). It is likewise often used in 3D cell cultures, where it promotes the proliferation and differentiation of various kinds of cells (Kim T. E. et al., 2016). PCL is a biocompatible and biodegradable polymer with adjustable hydrophobicity and cell adhesion abilities. Hydrophilicity can be enhanced by simple sodium hydroxide treatment (Bosworth et al., 2019). Cell adhesion and biocompatibility may be improved by coating with several of the aforementioned proteins (collagens, fibronectin, laminin, and RGD peptides) to mimic an HSC niche (Tallawi et al., 2015). In recent years, some experiments have been performed that lent support to the importance of PCL coating with proteins. Mousavi et al. (2019) coated PCL nanofibers with collagen I. Coating caused higher total cell counts (58 × 38-fold) and higher numbers of CD34^+^ cells (20-fold × 2.6-fold) compared with 2D cell culture. The ability to form colonies was significantly stronger in 3D cell culture based on the colony-forming assay. Fibronectin coating also has been shown to have a significant effect on HSC expansion. HSC cultured on fibronectin-coated PCL nanofibers have significantly greater expansion and expression of genes related to self-renewal. Greater expression of CD34 and CD45 markers in cells cultured on fibronectin-coated PCL nanofibers has been observed (Mousavi et al., 2018). Based on these findings, it can be assumed that PCL nanofiber coating promoted interactions between cells and provided a larger cell attachment area for cell expansion. In another comparative study, fibronectin-coated surface proved to have higher expansion potential in HSC and a higher percentage of CD34^+^ and CD45^+^ cells compared with when collagen I coating was used. Probably this is because fibronectin provided a larger surface for cell attachment and stronger adhesion forces in HSC (Kang et al., 2016). A supporting role of MSC with HSC has been proven in many cocultures on various materials. Total cell counts and percentages of CD34^+^ cells were significantly higher in coculture of HSC with MSC on PCL nanofibers compared with culture without MSC. The authors explained that the greater adhesion surface provided by MSC and thus higher proliferation rate of HSC contributed to greater HSC expansion (Ferreira et al., 2012). Moreover, the differentiation potential of HSC cultured on PCL nanofibers was also improved in comparison with 2D cell culture (Dehdilani et al., 2016). The results of current studies with PCL nanofibers point to positive effects on HSC self-renewal, differentiation, and migration. It is necessary, however, to coat PCL nanofibers with proteins and peptides, as well as to evaluate nanofiber thickness and scaffold pore size to ensure better biocompatibility.

Microfluidic cell cultures have been described for studying host–pathogen interactions (Barrila et al., 2018), and these systems can be enriched by PCL nanofibers scaffold to mimic various tissues. Based on this approach, pathogen-infected cells or pathogens with uninfected cells can be cultured to study host–pathogen interactions (Kim et al., 2019).

### Rotating Wall Vessel Culture

A rotating wall vessel (RWV) is a rotating cylinder filled with a cell culture medium where cells are constantly falling through the medium. In contrast to scaffold-based cell cultures, therefore, RWV cell culture allows cells to be in constant movement. Cells grown on culture plastic surfaces are collected and incubated with microcarrier beads for attachment (Nickerson et al., 2007). Microcarrier beads can be coated with ECM compounds, such as collagen (Radtke and Herbst-Kralovetz, 2012) or hyaluronic acid (Skardal et al., 2010). Cells attached to the beads are replaced with the RWV, and rotation is initiated. Within the RWV, cells can respond to chemical gradients and react with active molecules and microorganisms, and that means RWV cell culture can be used for cell differentiation and host–pathogen interaction studies. After an experiment, cells can easily be removed from the microbead carriers for further culture or evaluation (Nickerson et al., 2007). Pathogens can be added directly to the RWV or to the cell culture after recovery from RWV (Barrila et al., 2018).

### Organ-on-a-Chip Cell Culture

**FIGURE 3:**
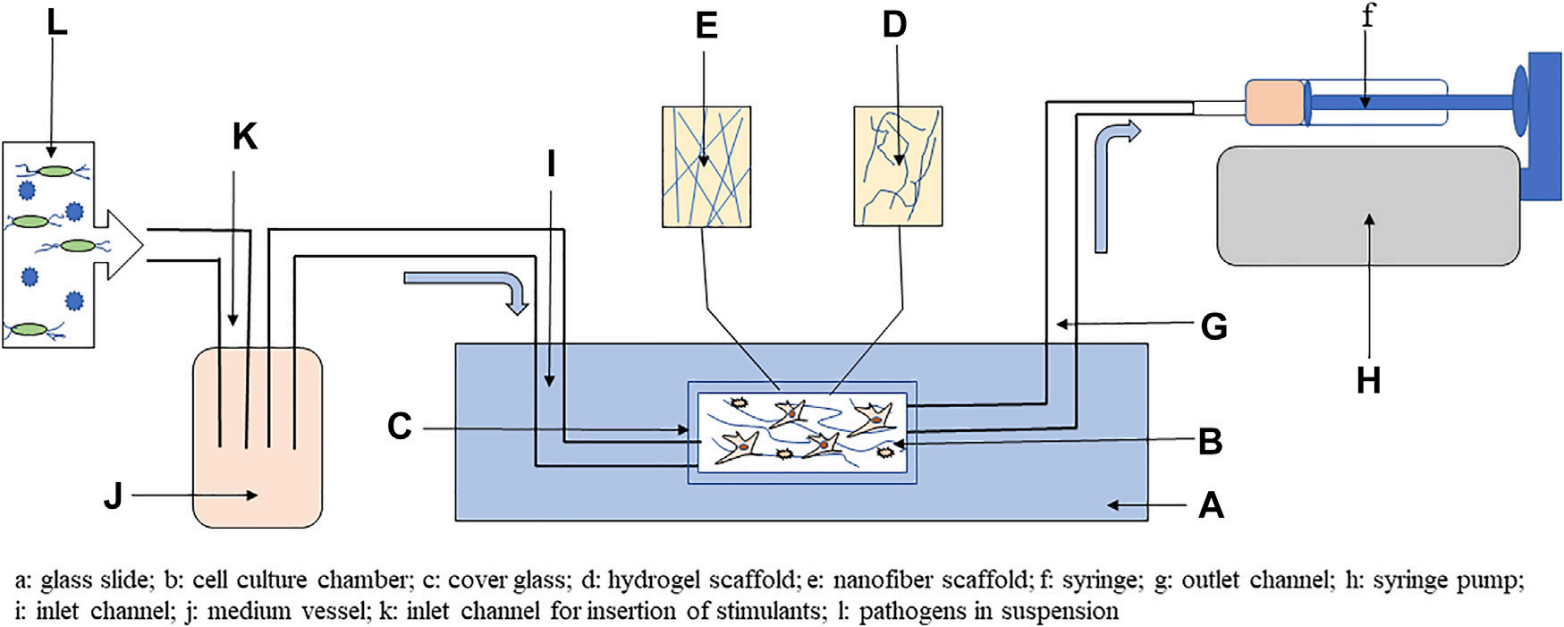
Simplified scheme using 3D cell culture with microfluidic system to study host–pathogen interactions. The main part of the system is a glass slide with chamber covered by glass coverslip, where hydrogels or nanofibers scaffold can be included. In the cell culture chamber, mesenchymal stem cells (MSC) and hematopoietic stem cells (HSC) can be cocultured on scaffold. The chamber is connected to a syringe by outlet channel, and flow of medium is produced by syringe pump through negative pressure. Cell culture medium is introduced into the chamber by inlet channel from the vessel. To study host–pathogen interactions, the medium vessel can be enriched via the inlet for inclusion of pathogens or other stimulants, such as metabolites, toxins, vitamins, or minerals. Adapted from Kim et al. (2019).

Dynamic processes in the bone marrow can be imitated by microfluidic technology based upon OCM. OCM consists of micro channels with a flowing medium separated by porous membranes that allow cells to remain in neighboring chambers (Barrila et al., 2018). In the case of HSC 3D culture preparation, the chambers can be filled with materials having a structure similar to that of the bone marrow. Furthermore, MSC can be precultured on chambers where they create an HSC niche by producing a stroma and such ECM components as fibronectin. HSC cultured on OCM have been found to remain in a primitive CD34^+^ state and be capable of differentiation and long-term culture for 28 days (Sieber et al., 2018). Similarly, microfluidic technology has been used to create a multigradient hydrogel system for HSC proliferation and differentiation assays within a 3D environment where surrounding ECM components and niche cells can be manipulated (Mahadik et al., 2014). For studying disease pathogenesis, disease causative agents can flow into the microchannels, and then cultured cells can be recovered and evaluated by morphological, genetic, and biochemical analyses (Kim H. J. et al., 2016). Figure 3 provides a schematic presentation as to the possible use of 3D HSC cell culture to study interactions with pathogens.

Polydimethylsiloxane (PDMS) is a commonly used polymer for OCM preparation and based on PDMS was developed 3D bone marrow on a chip with precultured MSC composed of two microchambers. In the top chamber, a coculture of MSC and HSC was performed, and the bottom chamber was separated by a porous membrane where the medium flows (Kefallinou et al., 2020). These membrane systems can be used to study host–pathogen interactions. *Staphylococcus aureus* infection model was used to study interactions with neutrophils, macrophages, and dendritic cells on poly(ε-caprolactone) nanofiber membrane. In this culture system, immune cells secreted TNF-α and IL-1α in comparison with 2D cell culture; therefore, 3D culture mimics standard inflammatory response as in the organism (Lee et al., 2021). The OCM culture can use scaffold-based cultures. Di Maggio et al. (2011) described a ceramic scaffold-based perfusion system with embedded MSC to mimic an HSC niche. Additionally, the platform allowed easy insertion of cytokines, growth factors, and potentially disease causative agents.

OCM cultures to study host–pathogen interactions were used mainly for mimicking respiratory and digestive tract disease pathogenesis. The importance of fluidic-based systems showed the study of Sunuwar et al. (2020). Luminal flow in their jejunal enteroid chip was able to stimulate the production of cyclic guanosine monophosphate upon exposure to heat-stable enterotoxin A from enterotoxigenic *Escherichia coli*. Similarly, Villenave et al. (2017) prepared microfluidic-based gut on chip and continuous flow enhanced viral replication and subsequently enterocyte damage. Mimicking physiological stretching of the gut caused by peristalsis can be an important factor for the increased invasion of some bacteria. For instance, *Shigella* human bacteria causing severe intestinal damage uses the peristaltic movement of the intestine wall and luminal flow to enhance their invasion potential (Grassart et al., 2019). The liver represents the most important organ for metabolic processes, and the liver on a chip is a valuable tool for studying the pathogenesis of hepatic diseases. Ortega-Prieto et al. (2018) prepared a microfluidic collagen scaffold-based liver on a chip with polarized primary human hepatocytes to examine the pathogenesis of hepatitis B virus, and they successfully imitated the production of cytokines and innate immune response as in the case of patients infected by hepatitis B virus.

OCM cultures were successfully used to imitate respiratory disease pathogenesis. Superinfection of influenza virus and *S. aureus* was observed in a virus–bacteria coculture on lung alveolus on chip (Deinhardt-Emmer et al., 2020). OCM cultures can play a role in models where it is impossible to study interactions *in vivo*. Surfactants on the surface of the respiratory tract create a part of a protective response against respiratory pathogens. Surfactant-deficient animals have high lethality; therefore, it is impossible to study pathogenesis in these animals. For this reason, Thacker et al. (2020) developed a lung on a chip with three layers, with the top layer composed of alveolar epithelial cells with macrophages with the bottom composed of endothelial cells and air–liquid interface. Through time-lapse imaging, they can reveal the dynamics of the initial phases of *Mycobacterium tuberculosis* infection and describe the host protective role of surfactants. The usability of a lung on a chip was also proved in viral infection models. Additionally, the impact of a lung on a chip devise on increased virulence of several serotypes of influenza virus was also proved (Si et al., 2019).

In a recent year, OCM is being developed to ensure that several probiotic bacteria strains are maintained for longer periods of time of more than 1 week; therefore, it can be used for studying the long-term effects of pathogens with the chronic progression of pathogenesis (Kim T. E. et al., 2016). Additionally, host–pathogen interactions using OCM cultures can be performed with various oxygen levels or under hypoxia, which is necessary for HSC quiescence (Shah et al., 2016).

### Using Three-Dimensional Cell Cultures to Study Host–Pathogen Interactions

**FIGURE 4:**
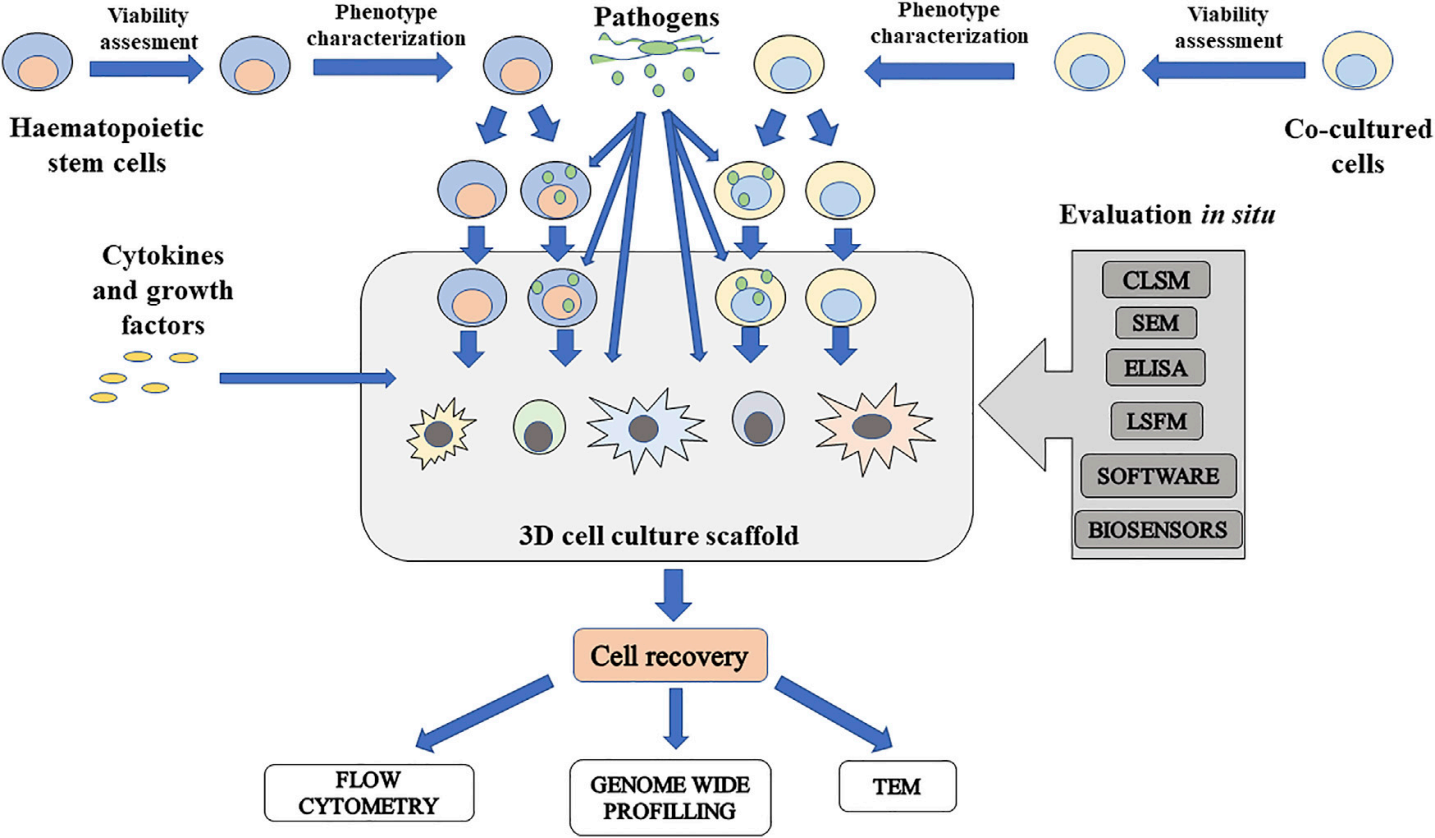
Schematic presentation of scaffold-based 3D cell culture to study influence of pathogens on hematopoiesis. Isolated hematopoietic stem cells (HSC) and cocultured cells can be infected before insertion to culture or after culturing in 3D culture. To study differentiation potential of infected HSC, cytokines and growth factors can be added and can initiate differentiation in various immune cells. At the same time, pathogens can be included to study how these disease causative agents can disrupt differentiation in immune cells. For *in situ* evaluation, there are some usable techniques. Imaging techniques include confocal laser scanning microscopy (CLSM), scanning electron microscopy (SEM), and light sheet fluorescence microscopy (LSFM). Content of molecules released by infected cells can be measured by standard ELISA method. Software-based techniques such as FluoroCellTrack can be used for evaluation of cells in flow, in microfluidic systems, or embedded cells in a scaffold. For monitoring of culture conditions, microchips for electrochemical detection of important measurable parameters such as oxygen, carbon dioxide, nutrients, and metabolite levels can be included. If it is possible to recover cells from the cultured scaffold, cells can be evaluated by antibody-based techniques, genome-wide profiling, and microscopy techniques such as transmission electron microscopy (TEM) for intracellular structure imaging.

The multipotency of HSC enables to differentiate them into various immune cells and study how pathogens interfere with hematopoiesis. The process of preparation includes cell seeding, the inclusion of cocultured cells, and growth factors and cytokines, which ensure HSC differentiation. Inclusion of pathogens can be performed in every step of the process, so this enables to study the disruption of hematopoiesis. The scheme of workflow for host–pathogen study using scaffold-based 3D stem cell cultures is described in Figure 4.

### Seeding of Cells

Cells can be seeded sequentially or simultaneously. It is better to seed them simultaneously because bone marrow HSC and MSC are located right next to each other. This system of cell seeding ensures homogenous localization (Baldwin et al., 2014). To mimic a bone marrow environment, scaffold-based cultures should be preferred. Then it is possible to seed cells on the upper sides of hydrogels or nanofiber scaffolds for cell migration studies. For the achievement of homogenous localization, cells can be mixed in liquid hydrogels. After the addition of crosslinker or through temperature-induced solidification, the creation of solid hydrogels is achieved (Rizwan et al., 2021). In the case of nanofiber scaffolds, cell localization is highly affected by pore size. Therefore, it is important to evaluate scaffold structure by scanning electron microscopy; otherwise, cell infiltration can be improved by sonication (Lee et al., 2011).

### Medium Exchange

Culture medium exchange is an important factor to mimic the bone marrow environment. After the exchange of the medium, cell culture losses cytokines and other substances produced by cocultured cells and pathogenic agents. On the other hand, the medium brings new nutrients to the culture. Low fluctuation in amounts of endogenic and exogenic factors entering the culture can be achieved by the exchange of low amounts of media in static cultures or a proper setup of a pump in microfluidic systems (Baldwin et al., 2014). The evaluation of stability in cell culture can be measured by biosensors in the culture because they can be used for the evaluation of oxygen, nutrients, pH, and metabolites. The problems of the current approaches in electrochemical sensing in 3D cultures are reviewed by Oliveira et al. (2021). In a dynamic microfluidic system, channel volumes and dilution requirements must be determined to obtain reliable results by detection systems (Castiaux et al., 2019). ELISA is used for the detection of molecules in a culture medium; therefore, it can be used for measurements of changes in metabolites (Kim et al., 2019).

### Evaluation *In Situ*

Standard morphological viability assays can be performed by fluorescence microscopy using confocal laser scanning microscopy (CLSM). CLSM can easily evaluate the location of cells and migration of cells in a scaffold by stacking images (Kim T. E. et al., 2016). The combination of CLSM with matrix-assisted laser desorption ionization-time (MALDI) proposes a complex analysis method. Machalkova et al. (2019) used CLSM to localize cells with fluorescence markers of apoptosis and proliferation. They also used MALDI for the detection of drugs of interest on tissue sections obtained from 3D cell culture. The principle of MALDI and usability in pathogen detection was reviewed by Singhal et al. (2015). Using CLSM can be problematic for scaffolds with higher thickness because of the loss of fluorescence signals of labelled molecules from deeper layers of more than 100 µm. On the other hand, light sheet fluorescence microscopy was able to create complete 3D tomography of tumor spheroids (Lazzari et al., 2019). Samples with thicknesses of more than 1 cm can be analyzed (Agrawal et al., 2021). After high-resolution images are obtained, the captured pictures can be analyzed by appropriate software equipment. Usable software-based analyses for 3D cell culture were reviewed by Agrawal et al. (2021). For instance, FluoroCellTrack is usable for high-throughput analyses of fluorescently labeled cells in a microfluidic system. Therefore, FluoroCellTrack can detect cells or droplets in a flow in a similar manner to flow cytometry but for longer periods of time (Vaithiyanathan et al., 2019). On the other hand, real-time monitoring of cells embedded in a scaffold can be performed by MetaXpress Software and ImageXpress Micro System, which allows to study the migration of cells toward the gradients (Agrawal et al., 2021).

### Cell Recovery

Another group of analytical methods comprises techniques after recovery of cells from 3D cell culture. However, the crucial step is cell recovery, which can cause serious problems, because the cell can be destroyed due to inappropriate treatment. Enzymatic cell recovery is a classic method for cell liberation from the hydrogel and nanofiber scaffolds. However, it must be taken with great care to avoid the degradation of cell receptors. The enzyme used for cell recovery is selected based on scaffold material hyaluronic acid (hyaluronidase), collagen (collagenase), et cetera (Caliari and Burdick, 2016). Cells cultured on nanofibers are usually collected by trypsinization (Kim T. E. et al., 2016; Kim et al., 2018). Enzyme concentration seems to be critical for achieving optimal cell liberation. Virumbrales-Muñoz et al. (2019) dealt with the optimization of cell recovery from collagen-based hydrogel in a microfluidic system. They tracked enzymatic degradation by confocal reflection microscopy, and the degradation rate was highly correlated with the concentration of the collagenase. For further experiments, collagenase’s highest concentration (8 mg/ml) was used and applied to the microfluidic system, and the scaffold was degraded. For 10 min, the majority of cells (80%) was extracted. This recovery method did not negatively affect the viability (above 90%) after cell recovery, and it was possible to reseed cells into another hydrogel. Cells were then used for gene expression analyses, and additionally cells were usable for image flow cytometry analyses, which support the usability of this approach as a gentle method to obtain cells from hydrogel scaffolds. Photodegradable PEG-based hydrogels propose more than easily degradable material for cell recovery. Shin et al. (2014) prepared photogel functionalized with anti-CD4 and anti-CD8 antibodies to isolate lymphocytes from a heterogenous cell population. Then, cell attachment sites were visualized by fluorescent microscopy; and then through site-specific exposure to UV light, CD4^+^ and CD8^+^ lymphocytes were successfully released. Isolating individual cells allows novel microscopy techniques, such as laser capture microdissection, which cut off the block from the scaffold, and subsequently, appropriate enzymes are used to digest scaffold and release cells (Oldenhof et al., 2020).

### Post-Harvest Evaluation of Cells

Post-harvest evaluation of cells in disease models comprises flow cytometry analyses of apoptotic and necrotic cells, phenotype markers, and activation markers in antigen-presenting cells. However, more comprehensive analyses can be performed through gene expression analyses of pro-inflammatory and anti-inflammatory cytokines, chemokines, receptors, proteins involved in programmed cell death, and pathogens (Kalaiyarasu et al., 2016). Genome-wide profiling of infected cells can provide deeper information about pathways involved in pathogen-induced immunosuppression (Lin et al., 2016). Various microscopy techniques can be used for the detection of pathogens, but precisely, the infection can be analyzed by transmission electron microscopy, which provides analyses of intracellular changes in response to infection (Rajput et al., 2014). The high-content single-cell technologies provide wide spectrum techniques to study host–pathogen interactions in different points of view, and all of them and their usability are reviewed by Chattopadhyay et al. (2018).

## Conclusion and Future Developments

The bone marrow as a source of immune cells in adult birds is disrupted by numerous diseases. Very little is known about these impacts in avian models. Immunosuppression caused by many avian diseases can be mitigated if we will have a better understanding of their pathogeneses, and therefore, we can create more effective vaccines and vaccination programs. That, in turn, will facilitate the realization of the genetic potential of poultry for maximum production while improving welfare in flocks. Poultry flocks are also a source of zoonotic diseases, and so preventing avian disease is very important to ensure human health globally. For these purposes, the creation of *in vitro* avian HSC niches for studying diseases’ pathogeneses can provide a valuable tool for improving global poultry health. Moreover, it can be used for studying pharmacokinetics and the effects of metabolites and additives on hematopoiesis. First, however, it is necessary to create an HSC niche *in vitro*. This review is a source of knowledge obtained from mammalian models that can be applied to achieve that objective.

## Glossary

2D: Two-dimensional
3D: Three-dimensional
Arteriolar endothelial cells
BC: B cells
BCP: B cell precursors
BG: Basophil granulocytes
CD4: CD4^+^ T cells
CD8: CD8^+^ T cells
CLP: Common lymphoid progenitors
CMP: Common myeloid progenitors
CLSM: Confocal laser scanning microscopy
CSF: Colony-stimulating factor
DC: Dendritic cells
DMEM: Dulbecco’s Modified Eagle’s Medium
DPBS: Dulbecco’s phosphate-buffered saline without Ca and Mg
ECM: Extracellular matrix
EG: Eosinophil granulocytes
GMP: Granulocyte-monocyte progenitors
HG: Heterophil granulocytes
HSC: Hematopoietic stem cells
HIF: Hypoxia-inducing factor
MA: Macrophages
MALDI: Matrix-assisted laser desorption ionization-time
MO: Monocytes
MODC: Monocyte-derived dendritic cells
MODM: Monocyte-derived macrophages
MSC: Mesenchymal stem cells
NK: Natural killer cells
OCM: Organ-on-a-chip
P: Plasmocytes
PAM: Polyacrylamide
PCL: Polycaprolactone
PDMS: Polydimethylsiloxane
PE: Phycoerythrin
PEG: Polyethylene glycol
RWV: Rotating wall vessel
SCF: Stem cell factor
SEC: Sinusoidal endothelial cells
TC: T cells
TCP: T cell precursors
YM: Young’s modulus
